# Bridging the Gap Between Research and Clinical Practice in Treatment Strategies and Mechanisms of Acute Ischemic Stroke

**DOI:** 10.3390/neurolint17040054

**Published:** 2025-04-08

**Authors:** Sonu M. M. Bhaskar

**Affiliations:** 1 Department of Neurology, Division of Cerebrovascular Medicine and Neurology, National Cerebral and Cardiovascular Center (NCVC), Suita 564-8565, Osaka, Japan; sonu.bhaskar@globalhealthneurolab.org; 2 Global Health Neurology Lab, Sydney, NSW 2150, Australia; 3 Ingham Institute for Applied Medical Research, Clinical Sciences Stream, Liverpool, NSW 2170, Australia; 4 NSW Brain Clot Bank, NSW Health Pathology, Sydney, NSW 2170, Australia; 5 Department of Neurology and Neurophysiology, Liverpool Hospital, South Western Sydney Local Health District and South West Sydney Clinical Campuses, UNSW Medicine, Liverpool, NSW 2170, Australia

Acute ischemic stroke (AIS) remains a formidable global health challenge, with approximately 11.9 million incident cases and 7 million deaths annually [1]. As the second leading cause of death worldwide and the third leading cause of death and disability combined, stroke imposes a staggering economic burden of over US$890 billion annually, projected to nearly double by 2050 [2]. This Special Issue, “*Treatment Strategy and Mechanism of Acute Ischemic Stroke*”, presents a timely update addressing critical aspects of AIS management, from innovative diagnostics to novel therapeutic strategies and mechanistic insights. By integrating findings, this Special Issue aims to bridge the gap between research and clinical practice, ultimately improving outcomes for the millions affected by stroke globally. This collection of studies represents a significant step towards a more personalized, mechanism-driven, and equitable approach to stroke care.

Imaging plays a pivotal role in AIS diagnosis, treatment selection, and prognostication [3,4]. Lakatos et al. demonstrated that pretreatment cranial computed tomography perfusion (CTP) can predict dynamic cerebral autoregulation changes post-recanalization therapy with 83% accuracy. Their findings highlight the potential of CTP-derived metrics, such as penumbra and infarct core volumes, to guide blood pressure management and predict outcomes. Similarly, Huang et al. proposed redefining infarction size for small-vessel occlusion using advanced imaging, offering a more precise classification that distinguishes small-vessel disease from branch atheromatous disease (BAD) with a sensitivity of 92% and specificity of 88%.

Understanding the mechanisms underlying AIS is essential for developing targeted therapies [5]. Wiszniewska et al. explored the role of Factor XIII in thrombolysis-treated patients, revealing its potential as a biomarker for stroke severity and prognosis. Sablić et al. investigated the synergistic effects of communicating arteries and leptomeningeal collaterals on patient outcomes. This study reinforces the important role of collateral circulation in determining stroke severity and recovery potential [6,7]. The recently proposed *Evolucollateral Dynamics* hypothesis, by our group, suggests that the genetic and environmental factors influencing collateral circulation have evolutionary underpinnings, which may impact stroke outcomes and therapeutic strategies [7]. Integrating this framework into future research could enhance our understanding of collateral remodeling and optimize treatment approaches for AIS. These mechanistic studies provide valuable insights into the complex interplay of vascular, hemostatic, and neuroprotective factors in AIS.

The development of novel therapeutic strategies is a cornerstone of AIS management. Drozdowska et al. evaluated the usability of a remote ischemic conditioning (RIC) device for pre-hospital stroke management, highlighting its potential to protect brain tissue during prolonged transport times. This study exemplifies the growing emphasis on early intervention and neuroprotection in AIS care. Additionally, Latacz et al. demonstrated the safety and efficacy of low-dose eptifibatide in patients with tandem occlusions, offering a promising pharmacological adjunct to endovascular therapy. Baki et al. highlight the life-saving role of decompressive surgery for malignant cerebellar infarction, reporting a 90-day mortality rate of 27.6% and good functional outcomes (mRS 0–3) in 41.4% of patients. Christidi et al. explore the potential of resting-state fMRI as a prognostic tool, demonstrating that increased amplitude of low-frequency fluctuation (ALFF) in bilateral M1 regions during subacute stroke is associated with better motor recovery. Another review by Crispino focuses on the dual complexities of hemorrhagic and ischemic events in patients with coagulation disorders, noting that direct oral anticoagulants can reduce major bleeding rates to as low as 2.13% annually compared to 3.09% with warfarin. Drakopanagiotakis et al. emphasize the critical need for respiratory rehabilitation, showing that respiratory muscle training can improve maximal inspiratory pressure (MIP) by 20–45 cm H_2_O and forced vital capacity (FVC) by 200–400 mL in stroke survivors. These improvements are associated with reduced respiratory complications and better functional outcomes. Together, these studies underscore the importance of personalized, mechanism-driven, and multidisciplinary approaches to optimize stroke care across the continuum [8].

Beyond acute settings, post-stroke rehabilitation remains a critical component of stroke management [8]. Baroni et al. reviewed the effectiveness of paired associative stimulation (PAS) in motor recovery, emphasizing its potential to enhance neuroplasticity and functional outcomes. Similarly, Lee and colleagues explored the application of muscle synergies for gait rehabilitation, providing a framework for personalized rehabilitation strategies. These studies highlight the importance of integrating advanced neurorehabilitation techniques into the continuum of stroke care [9].

Addressing disparities in stroke care delivery is essential for achieving equitable outcomes [10,11]. The studies in this issue underscore the need for systemic reforms to ensure access to advanced imaging, mechanical thrombectomy, and rehabilitation services, particularly in underserved regions [11]. Collaborative efforts between researchers, clinicians, and policymakers are crucial for overcoming these challenges.

To bridge the gap between research and clinical practice, an evidence-based, integrated framework for AIS management is essential. This framework emphasizes personalized diagnostics, mechanism-driven therapies, and comprehensive rehabilitation. Advanced imaging techniques, such as resting-state fMRI, can identify neuroplasticity patterns and guide individualized rehabilitation strategies [12], while biomarkers can inform surgical decision-making, as indicated by studies on decompressive surgery outcomes [13]. Mechanistically, understanding the interplay between coagulation disorders and ischemia [14,15], as highlighted by Crispino, facilitates targeted therapies that balance anticoagulation risks in complex patients. Comprehensive rehabilitation, including RMT, has demonstrated improvements in pulmonary function and functional mobility, as shown by Drakopanagiotakis et al. Moreover, equity in access to advanced diagnostics and therapies remains critical, particularly for underserved populations [10]. By integrating these dimensions, this framework aims to optimize outcomes and reduce disparities in stroke care.

This Special Issue highlights the urgent need for a paradigm shift in stroke care. While challenges such as standardizing methodologies and conducting larger-scale studies remain, the potential impact of these advancements is profound. With stroke burden expected to rise, particularly in low- and middle-income countries where 87% of stroke deaths occur [1], implementing these evidence-based strategies is not just a public health or scientific imperative but a moral one. In conclusion, by fostering global collaboration, prioritizing patient-centered approaches, and addressing healthcare disparities [16], we can translate these research insights into tangible improvements in stroke outcomes worldwide. The time for action is now—building on past consensus efforts, including the European Stroke Action Plan (2018–2030), which sets clear targets to reduce stroke incidence, improve care, and implement evidence-based strategies, with millions of lives relying on our collective commitment [17].
